# Поздравляем с юбилеем, дорогая Валентина Александровна!

**DOI:** 10.14341/probl12856

**Published:** 2021-12-30

**Keywords:** Валентина Александровна Петеркова, детская эндокринология, юбилей

## Abstract

1 декабря 2021 года — юбилей у Валентины Александровны Петерковой, профессора, академика РАН, научного руководителя Института детской эндокринологии ФГБУ «НМИЦ эндокринологии» Минздрава России, главного внештатного специалиста детского эндокринолога Минздрава России, заслуженного врача Российской Федерации.

## Abstract (en)

December 1, 2021 — the anniversary of Valentina Aleksandrovna Peterkova, professor, academician of the Russian Academy of Sciences, scientific director of the Institute of Pediatric Endocrinology of the National Research Center of Endocrinology of the Ministry of Health of Russia, chief freelance specialist of the pediatric endocrinologist of the Ministry of Health of Russia, Honored Doctor of the Russian Federation.

От всей души поздравляем с юбилеем любимого учителя, талантливого врача, великолепного педагога и профессионала высочайшего уровня!

1 декабря 2021 года — юбилей у Валентины Александровны Петерковой, профессора, академика РАН, научного руководителя Института детской эндокринологии ФГБУ «НМИЦ эндокринологии» Минздрава России, главного внештатного специалиста детского эндокринолога Минздрава России, заслуженного врача Российской Федерации.

Жизненный и профессиональный путь Валентины Александровны может служить для молодежи примером того, как стать грамотным высококвалифицированным врачом и талантливым ученым. Успешно окончив в 1966 г. лечебный факультет 2-го Московского медицинского института им. Н.И. Пирогова, Валентина Александровна Петеркова поступила в ординатуру Института педиатрии АМН СССР, а позже, в 1969 г., в аспирантуру на кафедру детских болезней 2-го МОЛГМИ, где получила глубокую и основательную врачебную подготовку.

Проработав на кафедре детских болезней 2-го МОЛГМИ в течение 19 лет, Валентина Александровна последовательно прошла путь от ассистента до профессора кафедры, успешно защитив в 1989 г. докторскую диссертацию. Вся трудовая биография Петерковой В.А. связана с педиатрией и детской эндокринологией.

С 1990 г. профессиональная деятельность Петерковой Валентины Александровны связана с Эндокринологическим научным центром (ныне ФГБУ «НМИЦ эндокринологии» Минздрава России). Более 30 лет Валентина Александровна возглавляет детскую клинику Эндокринологического научного центра, реорганизованную при ее активном участии в 2002 г. в Институт детской эндокринологии, научным руководителем которого она и является в настоящее время. На занимаемой должности ярко проявились великолепный организаторский талант и неординарные способности Валентины Александровны.

Валентине Александровне Петерковой удалось объединить многие научные коллективы для решения фундаментальных проблем по всем ключевым направлениям эндокринопатий детского возраста, разработке вопросов диагностики, лечения, профилактики и мониторинга различных заболеваний эндокринной системы у детей и подростков. Валентина Александровна участвовала в разработке и внедрении молекулярно-генетических методов диагностики эндокринных заболеваний у детей, создании программ медико-генетического консультирования семей, имеющих детей с сахарным диабетом, врожденной дисфункцией коры надпочечников и другими эндокринопатиями.

Фундаментальные и прикладные исследования В.А. Петерковой касаются практически всех вопросов клинической эндокринологии детского возраста. В.А. Петерковой принадлежит приоритет в изучении нарушений секреции гормона роста при различных заболеваниях эндокринной системы у детей. Под руководством В.А. Петерковой разработаны дифференциально-диагностические алгоритмы диагностики и лечения различных форм низкорослости у детей, внедрена методика ростостимулирующей терапии при дефиците гормона роста; разработаны и внедрены принципы скрининга новорожденных на врожденный гипотиреоз и врожденную дисфункцию коры надпочечников в Российской Федерации. Важным направлением научной деятельности В.А. Петерковой является изучение вопросов диагностики и лечения различных опухолей гипоталамо-гипофизарной области в детском возрасте. В.А. Петеркова внесла большой вклад в изучение этиологии, патогенеза и профилактики сахарного диабета у детей и подростков. Большое внимание уделяет В.А. Петеркова редким заболеваниям, в том числе диагностике и лечению неонатального сахарного диабета, аутоиммунного полигландулярного синдрома 1 типа, синдрома Мак-Кьюна–Олбрайта–Брайцева, врожденного гиперинсулинизма и других.

В.А. Петеркова — главный внештатный специалист детский эндокринолог Минздрава России, работая в этом качестве, она сумела объединить региональные медицинские учреждения и научные коллективы для решения вопросов, связанных с оказанием специализированной медицинской помощи детям и подросткам с эндокринными заболеваниями, лекарственным обеспечением, санаторно-курортным лечением. Под руководством В.А. Петерковой разработаны, внедрены и регулярно актуализируются клинические рекомендации по детской эндокринологии.

В.А. Петеркова — заведующая кафедрой детской эндокринологии-диабетологии Института ВДПО ФГБУ «НМИЦ эндокринологии» Минздрава России, где ежегодно повышают свою квалификацию свыше 250 детских эндокринологов из разных регионов России. Под руководством В.А. Петерковой защищено 18 докторских и свыше 50 кандидатских диссертаций. В.А. Петеркова — автор 400 научных публикаций, в том числе 10 монографий.

В.А. Петеркова — член Европейского общества педиатров-эндокринологов (ESPE) и Европейского общества детских диабетологов (ESPAD), член правления Московского общества детских врачей и Московской городской ассоциации эндокринологов, член Президиума Правления Российской ассоциации эндокринологов.

На протяжении многих лет В.А. Петеркова возглавляла Российскую диабетическую ассоциацию (РДА) и сумела не только сплотить региональные общества пациентов с сахарным диабетом, но и сделать очень многое в повышении доступности и качества медицинской помощи пациентам с сахарным диабетом. Именно Валентина Александровна придумала проведение ежегодных Диаспартакиад для детей с сахарным диабетом 1 типа. Безусловно, все это требует ежедневной душевной отдачи и огромных физических сил.

В.А. Петеркова долгие годы была заместителем главного редактора журнала «Проблемы эндокринологии» и очень много сделала для развития и продвижения этого издания, является членом редколлегии журналов «Педиатрия», «Российский педиатрический журнал», заместителем председателя диссертационного Ученого совета ФГБУ «НМИЦ эндокринологии» Минздрава России.

В 2012 г. Валентина Александровна избрана членом-корреспондентом РАН, в 2016 г. — академиком РАН.

В.А. Петеркова награждена орденом «Дружбы», медалью «В память 850-летия Москвы», медалью «За заслуги перед отечественным здравоохранением».

Валентина Александровна — настоящий детский доктор, талантливый организатор и чудесный человек! Умение грамотно спланировать свой рабочий график, руководить одновременно десятками проектов, вести консультативную работу, регулярно проводить профессорские обходы и, самое главное, — за ворохом бумажных дел не забывать интересы больного ребенка и его родителей. На это способен только глубоко преданный своему делу человек!

В день Вашего юбилея примите от всех нас, Ваших учеников, коллег, друзейи благодарных пациентов и их родителей, самые искренние и душевные поздравления!Желаем Вам крепкого здоровья, неиссякаемого оптимизма, исполнения задуманного,достойных учеников, всего самого доброго!

